# New Chitosan-Based Co-Delivery Nanosystem for Diabetes Mellitus Therapy

**DOI:** 10.3390/polym16131825

**Published:** 2024-06-27

**Authors:** Florentina Geanina Lupascu, Alexandru Sava, Simona-Maria Tătărușanu, Andreea-Teodora Iacob, Andrei Dascălu, Bianca-Ștefania Profire, Ioana-Mirela Vasincu, Maria Apotrosoaei, Tudor-Cătălin Gîscă, Ioana-Andreea Turin-Moleavin, Lenuta Profire

**Affiliations:** 1 Department of Pharmaceutical Chemistry, Faculty of Pharmacy, “Grigore T. Popa” University of Medicine and Pharmacy of Iași, 16 Universitaty Street, 700115 Iași, Romania; florentina-geanina.lupascu@umfiasi.ro (F.G.L.); tatarusanu.simona-maria@email.umfiasi.ro (S.-M.T.); andreea.panzariu@umfiasi.ro (A.-T.I.); ioana-mirela.vasincu@umfiasi.ro (I.-M.V.); apotrosoaei.maria@umfiasi.ro (M.A.); lenuta.profire@umfiasi.ro (L.P.); 2 Department of Analytical Chemistry, Faculty of Pharmacy, “Grigore T. Popa” University of Medicine and Pharmacy of Iași, 16 University Street, 700115 Iași, Romania; alexandru.i.sava@umfiasi.ro; 3 Research & Development Department, Antibiotice Company, 1 Valea Lupului Street, 707410 Iași, Romania; 4 Centre of Advanced Research in Bionanoconjugates and Biopolymers, “Petru Poni” Institute of Macromolecular Chemistry, 41A Grigore Ghica-Voda Alley, 700487 Iași, Romania; idascalu@icmpp.ro (A.D.); moleavin.ioana@icmpp.ro (I.-A.T.-M.); 5 Department of Internal Medicine, Faculty of Medicine, “Grigore T. Popa” University of Medicine and Pharmacy of Iași, 16 University Street, 700115 Iași, Romania; 6 Faculty of Medicine, “Grigore T. Popa” University of Medicine and Pharmacy of Iasi, 16 University Street, 700115 Iași, Romania; gisca_tudor-catalin@d.umfiasi.ro

**Keywords:** curcumin, pioglitazone, chitosan, co-delivery, nanoparticles, HPLC

## Abstract

Type 2 diabetes mellitus (T2DM) is one of the most common metabolic disorders, with a major involvement of oxidative stress in its onset and progression. Pioglitazone (Pio) is an antidiabetic drug that mainly works by reducing insulin resistance, while curcumin (Cur) is a powerful antioxidant with an important hypoglycemic effect. Both drugs are associated with several drawbacks, such as reduced bioavailability and a short half-life time (Pio), as well as instability and poor water solubility (Cur), which limit their therapeutic use. In order to overcome these disadvantages, new co-delivery (Pio and Cur) chitosan-based nanoparticles (CS-Pio-Cur NPs) were developed and compared with simple NPs (CS-Pio/CS-Cur NPs). The NPs were characterized using dynamic light scattering (DLS), transmission electron microscopy (TEM), X-ray diffraction (XRD), and Fourier-transform infrared spectroscopy (FTIR). In addition, the entrapment efficiency (EE) and loading capacity (LC), as well as the release profile, of the APIs (Pio and Cur) from the CS-APIs NPs in simulated fluids (SGF, SIF, and SCF) were also assessed. All the CS-APIs NPs presented a small particle size (PS) (211.6–337.4 nm), a proper polydispersity index (PI) (0.104 and 0.289), and a positive zeta potential (ZP) (21.83 mV–32.64 mV). Based on the TEM results, an amorphous state could be attributed to the CA-APIs NPs, and the TEM analysis showed a spherical shape with a nanometric size for the CS-Pio-Cur NPs. The FT-IR spectroscopy supported the successful loading of the APIs into the CS matrix and proved some interactions between the APIs and CS. The CS-Pio-Cur NPs presented increased or similar EE (85.76% ± 4.89 for Cur; 92.16% ± 3.79 for Pio) and LC% (23.40% ± 1.62 for Cur; 10.14% ± 0.98 for Pio) values in comparison with simple NPs, CS-Cur NPs (EE = 82.46% ± 1.74; LC = 22.31% ± 0.94), and CS-Pio NPs (EE = 93.67% ± 0.89; LC = 11.24% ± 0.17), respectively. Finally, based on the release profile results, it can be appreciated that the developed co-delivery nanosystem, CS-Pio-Cur NPs, assures a controlled and prolonged release of Pio and Cur from the polymer matrix along the GI tract.

## 1. Introduction

Diabetes mellitus (DM), mainly represented by type 2 diabetes, accounting for more than 90% of all cases, is a chronic metabolic disorder characterized by persistent hyperglycemia, resulting from decreased insulin secretion, insulin resistance, or both [1,2]. The global prevalence of DM, which is considered one of the five main causes of death worldwide, was 537 million in 2021 and is predicted to increase during the next years because of the careless lifestyle of people [3]. Also, it is worrying that the incidence of DM among children and teenagers is increasing [4]. Moreover, chronic hyperglycemia is associated with micro- and macrovascular complications, leading to blindness, kidney failure, heart disease, stroke, and gangrene, which often requires foot amputation [5].

Nowadays, there is much evidence that oxidative stress plays an important role in the pathogenesis of DM by decreasing the proliferation of pancreatic β-cells, which contributes to the impairment of insulin secretion. Normally, the body produces endogenous antioxidants (e.g., superoxide dismutase, catalase, glutathione peroxidase, and glutathione S-transferase) that scavenge excess reactive oxygen species (ROS) and protect against several diseases, such as DM, cancer, and heart disease [6].

To ensure its success, antidiabetic therapy should target two important aspects: (i) optimal glycemic control, as soon as possible, in order to reduce the impact of glucose toxicity and (ii) the proper control of associated risk factors, including oxidative stress, dyslipidemia, mitochondrial dysfunction, and micro/macrovascular complications [7].

It is also known that the micro/macrovascular complications of DM are closely associated with oxidative stress and inflammation [8]. Consequently, drugs with hypoglycemic, antioxidant, and anti-inflammatory effects could be a good option for DM management, and the use of combined therapy has recently been taken into consideration.

Pioglitazone (Pio) is a reference antidiabetic drug, usually indicated at a dose of 15–30 mg/day, which belongs to the thiazolidine-2,4-dione (TZD) class and mainly works by reducing insulin resistance. It acts as a peroxisome proliferator-activated receptor (PPAR-γ) agonist that modifies the transcription of some genes involved in glucose and lipid metabolism [8]. In addition, Pio inhibits the expression of proinflammatory proteins such as cyclooxygenase-2 (COX-2), inducible nitric oxide synthase (iNOS), and several cytokines. There is also evidence that supports that Pio decreases oxidative stress in the pancreatic β-cells of DM mice and improves their function, even if the level of PPAR-γ receptors is low [8]. Despite its notable biological effects, the use of Pio in T2DM treatment is limited because it has a very limited water solubility (0.039 mM), low bioavailability, a short half-life of 3–6 h, and a fast clearance rate. In order to overcome these disadvantages, numerous studies have focused on increasing its bioavailability and half-life using polymeric nanocarriers, like nanoparticles (NPs) [9].

NPs, as with other delivery systems like inclusion nanofibers, have been intensively investigated as co-drug delivery systems with different properties [10,11]. In the case of NPs, the most used methods for co-encapsulation are physical and chemical (e.g., covalent chemical bonds between polymers and drugs) co-encapsulation [10]. NPs protect the loaded drugs from the acidic environment of the gastrointestinal tract (GI) and increase the drug bioavailability by promoting the crossing of the GI epithelium via transcellular or paracellular transport pathways [12].

Curcumin (Cur) is the main curcuminoid that is mainly extracted from the rhizome of *Curcuma longa*. Numerous studies have shown that Cur has numerous biological effects, such as antioxidant, hypoglycemic, antimicrobial, anti-inflammatory, anti-cancer, anti-angiogenic, and neuro- and cardio-protective effects, as well as anti-obesity properties. The effectiveness of Cur is limited due to its instability and poor solubility in water (0.6 µg/mL), with doses ranging between 500 and 2000 mg/day [13,14]. Also, in biological conditions, it is rapidly metabolized, the main sites being the liver as well as the intestine through the microbiota. A maximum of 60 mg/mL of Cur was measured in rat serum, even after oral administration of 500 mg/kg [15,16]. To improve its stability, solubility, and bioavailability, different nanoformulations, such as solid lipid NPs, nanogels, cyclodextrin complexes, and nanoemulsions, have been developed [17].

Chitosan (CS) is one of the most used polymers for nanocarrier formulations. It is a natural polymer derived from chitin that possesses several beneficial properties, such as antibacterial activity, non-toxicity and biocompatibility, biodegradability, and permeability [18].

Based on the hypoglycemic effect of Pio and the antioxidant properties of Cur, new co-delivery CS-based NPs loaded with Cur and Pio as active pharmaceutical ingredients (APIs) were developed using the physical co-encapsulation method. This multitarget co-delivery nanosystem (CS-Pio-Cur NPs) was fully physicochemically characterized using dynamic light scattering (DLS), transmission electron microscopy (TEM), X-ray diffraction (XRD), and Fourier-transform infrared spectroscopy (FTIR), in comparison with simple nanosystems (CS-Pio/CS-Cur NPs), in order to highlight the advantages of the co-delivery formulation. In addition, the entrapment efficiency (EE) and loading capacity (LC) were also assessed using the UV spectrophotometric method. Finally, the release profile of the APIs (Pio and Cur) from the CS-APIs NPs was studied, and to quantify Pio and Cur, a high-performance liquid chromatography (HPLC) method was developed and validated.

## 2. Materials and Methods

### 2.1. Materials

All chemicals used in this research were of analytical grade or HPLC p.a. quality, certified by commercially available sources, and were used without further purification unless otherwise specified. Curcumin (≥94% curcuminoid content; ≥80% curcumin), pioglitazone hydrochloride (MW = 392.90 g/mol), low-molecular-weight chitosan (MW = 50–190 kDa, 75–85% deacetylation degree, and 20–300 cP viscosity), pentasodium tripolyphosphate (TPP), acetic acid (min. 99.8%; p.a. ACS reagent), and Tween 80 were purchased from Sigma Aldrich (St. Louis, MO, USA).

### 2.2. Preparation of CS-APIs NPs

In order to find the best formulation, the CS NPs loaded with different concentrations of the APIs (Cur, Pio, and a Cur–Pio combination) were developed, and their preparation was reported in our previous work [19]. Briefly, to 3 mL of 0.1% (*w*/*v*) CS, different concentrations of the APIs (Pio: 0.6 mg/0.5 mg/0.4 mg/0.3 mg; Cur: 1.5 mg/1.3 mg/1.2 mg/1.0 mg; or Pio-Cur: 0.6–1.5 mg/0.5–1.3 mg/0.4–1.2 mg/0.3–1.0 mg) dissolved in 0.5 mL of ethanol were added. Then, 1 mL of a 0.1% (*w*/*v*) TPP aqueous solution was added dropwise under stirring, and CS-APIs NPs were obtained. The samples were washed several times (until the APIs were not detected in the wash water) and centrifuged, and the resulting pellets were resuspended in distilled water and then lyophilized to obtain CS-APIs NP powders.

### 2.3. Characterization of CS-APIs NPs

#### 2.3.1. The Physical Parameters

The particle size (PS) and the polydispersity index (PI) measurements of the developed CS-based NPs (CS-Pio-Cur NPs, CS-Pio NPs, and CS-Cur NPs) were determined by dynamic light scattering (DLS) using an Easier Nano ZS90 instrument (Malvern Instruments, Malvern, UK) with a scattering angle 90°. The zeta potential (ZP) measurements were also performed using the same instrument employing the electrophoretic mobility of the NP suspensions. Each NP formulation was suspended in distilled water at room temperature, homogenized at 10,000 rpm, and then transferred into a particle-sizing cell. All measurements were performed in triplicate.

#### 2.3.2. Transmission Electron Microscopy (TEM) analysis

Transmission electron microscopy (TEM) micrographs were obtained using a Hitachi High-Tech HT7700 transmission electron microscope (Hitachi High Technologies America, Inc., Schaumburg, IL, 60173 USA) operating at a 100 kV accelerating voltage in high-contrast mode. The NP samples were placed on carbon-coated copper grids with a mesh size of 300 and dried at room temperature, and then the images were recorded.

#### 2.3.3. X-ray Diffraction

The physical states (crystalline or amorphous) of the APIs (Cur, Pio, and Pio-Cur) unloaded and loaded into CS-APIs NPs (CS-Cur NPs, CS-Pio NPs, and CS-Pio-Cur NPs) were analyzed using a Rigaku SmartLab X-ray diffractometer (Rigaku Corporation, Tokyo, Japan) in Bragg–Brentano geometry with a Cu anode (with an X-ray wavelength of 1.5406 Å) in an angular range of 2–50°, with a scanning step of 0.02° and a recording rate of 3°/min.

#### 2.3.4. Fourier-Transform Infrared Spectroscopy

The Fourier-transform infrared (FTIR) spectra of the APIs (Cur, Pio, and the Pio-Cur physical mixture) and the CS-APIs NPs (CS-Cur NPs, CS-Pio NPs, and CS-Pio-Cur NPs) were recorded using an FTIR spectrophotometer (ABB-MB3000 FT-IR MIRacleTM Single Bounce ATR—cristal ZnSe) (ABB, Zurich, Switzerland). The spectra processing was carried out with the Horizon MB™ FT-IR software (Horizon MBTM FT-IR software 3.1.29.5.). A scanning range of 400–4000 cm^−1^ with 32 scans with a resolution of 4 cm^−1^ was applied.

#### 2.3.5. Entrapment Efficiency (EE) and Loading Capacity (LC)

The developed CS-APIs NPs, CS-Cur NPs (a: 1.5 mg, b: 1.3 mg, c: 1.2 mg, and d: 1.0 mg), CS-Pio NPs (a: 0.6 mg, b: 0.5 mg, c: 0.4 mg, and d: 0.3 mg), and CS-Pio-Cur NPs (a: 0.6 mg–1.5 mg, b: 0.5 mg–1.3 mg, c: 0.4 mg–1.2 mg, and d: 0.3 mg–1.0 mg), were studied in terms of their EE (%) and LC (%) in order to establish the optimal formulation.

The EE (%) of the APIs (Cur and Pio) into the CS NPs was quantified using a UV spectrophotometric method (UVIKNO XL, BIOTECH Instruments (BioTek Instruments, Winooski, VT, United States). After the separation of the CS-APIs NPs, the supernatant (TPP solution) was centrifuged at 15,000 rpm for 30 min at 4 °C and then passed through a 0.22 µm filter. The absorbance of the filtrate was measured at 424 nm (for Cur) and 272 nm (for Pio) [14]. In order to quantify the concentrations of the APIs, the standard curves for Pio and Cur using different concentrations, ranging from 9.9 to 47.619 μg/mL for Pio (R^2^ = 0.999) and 2.38 to 10 μg/mL for Cur (R^2^ = 0.999), were plotted. The EE (%) was calculated using the following equation:

EE (%) = C_1_/C_0_ × 100 (1)

where

C_0_ = the initial concentration of the APIs (Cur and Pio);
C_1_ = the concentration of the APIs, measured in the TPP supernatant.

The LC (%) of the APIs into the CS NPs was measured based on the method reported in the literature [20]. A quantity of freeze-dried CS-APIs NPs (12 mg of CS-Pio NPs, 13 mg of CS-Cur NPs, and 14 mg of CS-Pio-Cur NPs) was treated with ethanol (10 mL). The mixture was sonicated for 5 min, and after that, it was centrifuged for 10 min at 15,000 rpm at 4 °C. Then, the supernatant was used to measure the absorbance at 424 nm (for Cur) and 272 nm (for Pio) [21,22]. The LC (%) was calculated using the following equation:

(2) $$\mathrm{LC}\;(\%)=\frac{wAPIs}{wCS-APIs\;NPs}\times 100$$

where

wAPIs = the quantity of the APIs (Cur and Pio) loaded into the CS NPs;
wCS-APIs NPs = the quantity of CS-APIs NPs.

### 2.4. The Study of APIs’ Release from CS-APIs NPs

#### 2.4.1. Development and Validation of HPLC Method

To quantify the APIs (Pio, Cur) released from the CS-based NPs (CS-Cur/CS-Pio/CS-Pio-Cur NPs), an HPLC method was validated using a Shimadzu Nexera LC-40-XR system (Shimadzu, Kyoto, Japan) equipped with a serial dual-plunger pump, an autosampler (SIL 40 XR), an SPD-40V series UV-Vis, and an RF-20Axs fluorescence detector. Chromatographic separation of the APIs was performed in a C18 column (2.1 × 100 mm, Waters CORTECS 2.7 μm) using two mobile phases: A (water/formic acid—99.9/0.1, *v*/*v*) and B (tetrahydrofuran). Before use, the solvents were filtered through a 0.22 μm filter and degassed by ultrasonication. The injection sample amount was 10 μL, the run time was 20 min in isocratic mode (0.6 mL/min), and the optimal mobile phase ratio was A (62%) to B (38%). The column temperature was kept at 30 °C during the chromatographic operation with UV-Vis detection for Pio (270 nm) and fluorescence detection for Cur (λ_ex_ = 420 nm and λ_em_ = 550 nm).

The qualitative and quantitative analysis of the APIs (Pio and Cur) was carried out based on the retention times and peak areas, respectively. The LabSolutionDB software 6.106SP1 was used for the peak integration.

*Plotting calibration curves for APIs*

To plot the calibration curves, stock solutions (containing 2000 ppm) of the standard APIs (Pio and Cur) were prepared by dissolving 50.0 mg of Pio or Cur in 25 mL of acetonitrile. From the stock solutions, serial dilutions (0.5, 1.5, 2.5, 5, 10, 20, 40, 60, 80, and 100 ppm of Pio or Cur) were prepared. The experiments were performed in triplicate.

A calibration curve is a plot of the area under the peak (AU) to the external standard as a function of the drug concentration:

AU = Slope × Concentration + Intercept(3)

The slope and the intercept were determined from the AU and the concentration of the APIs. Using this equation, the Cur and Pio released from the CS-based NPs (CS-Cur/CS-Pio/CS-Pio-Cur NPs) were quantified.

#### 2.4.2. In Vitro API Release Model

To study the release of the APIs (Cur and Pio) from the CS-APIs NPs, an in vitro digestion model was used. The release study was performed using simulated gastric fluid (SGF (pH 1.6)), simulated intestinal fluid (SIF (pH 7.0)), and simulated colonic fluid (SCF (pH 7.4)) (Table 1).

A quantity of CS-APIs NPs—13 mg of CS-Pio-Cur NPs (containing 2.66 mg of Cur and 0.38 mg of Pio), 9 mg of CS-Cur NPs (containing 2.47 mg of Cur), and 8 mg of CS-Pio NPs (containing 0.38 mg of Pio)—was mixed with 50 mL of SGF (pH 1.6), SIF (pH 7.0), and SCF (pH 7.4), respectively. Each mixture was then shaken at 80 rpm at 37 °C, and every 30 min, during 3 h, 300 μL of each sample was collected and centrifuged for 10 min at 15,000 rpm at 4 °C. The supernatants were filtered through a 0.22 μm filter, and then 10 μL of each sample was injected using HPLC method conditions. The experiment was performed in triplicate.

The concentration (%) of the APIs (Pio and Cur) released from the NPs into the simulated fluids (SGF, SIF, and SCF) was calculated using the following equation [14]:

(4) $$APIs\;Released\;(\%)=\frac{C_{1}}{C_{0}}\times 100\%$$

where

C_1_ = the concentration of the APIs (Cur and Pio) released into the simulated fluids (SGF, SIF, and SCF);
C_0_ = the concentration of the APIs loaded into the CS-based NPs.

#### 2.4.3. Statistical Analysis

The results were expressed as mean values ± standard deviation (SD), and the analysis was performed using IBM SPSS Statistics 23.0.0.0 for Windows. The statistical significance of the results was assessed by one-way and two-way analyses of variance (the ANOVA test) followed by Tukey’s HSD test, which was used to compare the differences between samples. A *p*-value of ˂0.05 was considered statistically significant.

## 3. Results and Discussion

### 3.1. Characterization of CS-APIs NPs

#### 3.1.1. The Physical Parameters

The developed CS-APIs NPs were characterized in terms of their particle size (PS), polydispersity index (PI), and zeta potential (ZP), and the results are presented in Table 2.

PS is a key parameter for the characterization of drugs and drug delivery systems in terms of their stability, absorption, release, and biodistribution. All CS-APIs NPs presented a small PS, ranging between 211.6 and 337.4 nm. In addition, a proper PI (ranging between 0.104 and 0.289) and a positive ZP value (21.83 mV–32.64 mV) were also recorded. It is known that a zeta potential within a limit of ± 30 mV and higher is an important parameter indicating the stability of NPs [23]. In the case of the co-delivery of CS-Pio-Cur NPs, the ZP values ranged between 29.03 mV (formulation a) and 32.64 (formulation d).

#### 3.1.2. Transmission Electron Microscopy (TEM) Analysis

**Figure 1:**
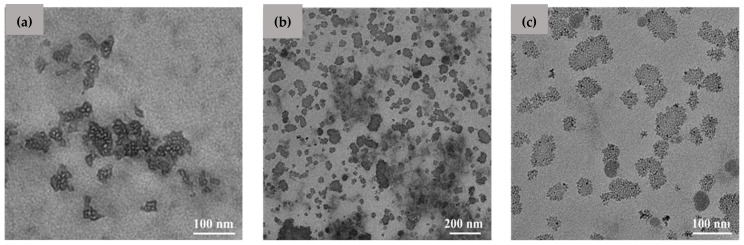
TEM images of CS NPs (**a**) and CS-Pio-Cur NPs (**b**,**c**).

The micrographs of the CS-Pio-Cur NPs in the reference cu CS NPs are presented in Figure 1. It was observed that the CS-Pio-Cur NPs had an almost spherical shape with a nanometric size (Figure 1b,c) and were more homogeneous in terms of their dimensions in comparison with the CS NPs (Figure 1a).

#### 3.1.3. X-ray Diffraction

**Figure 2:**
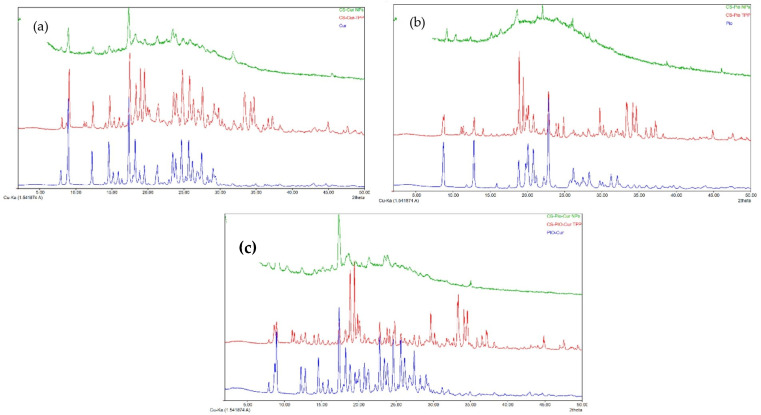
XRD spectra of CS-APIs NPs ((**a**): CS-Cur, (**b**): CS-Pio, and (**c**): CS-Pio-Cur NPs) in comparison with Cur, Pio, and physical mixtures of Pio-Cur and CS-APIs-TPP.

The crystalline state of APIs influences their stability, solubility, and bioavailability. The XRD spectra of the CS-APIs NPs (CS-Cur NPs, CS-Pio NPs, and CS-Pio-Cur NPs) in comparison with the APIs (Cur, Pio, and the Cur-Pio physical mixture) are presented in Figure 2. In addition, the XRD spectra of the CS-APIs-TPP physical mixture are presented for comparison.

The analysis of the APIs’ diffractograms revealed that the Cur, Pio, and Cur-Pio physical mixture were in a crystalline state, their diffraction profiles being confirmed through the reported data [24,25]. Cur (Figure 2a) presents strong and sharps diffraction peaks at 2θ angles of 9°, 12.26°, 14.58°, 17.36°, and 18.24° and more peaks in the angle range of 20° to 30°. Referring to Pio (Figure 2b), multiple obvious sharp peaks were observed at 2θ angles of 8.7°, 12.8°, 18.82°, and 22.82°. The crystalline structure of Cur and Pio was preserved in the CS-APIs-TPP physical mixture (Figure 2c).

In the case of the CS-APIs NPs, a significant modification of the crystalline structure was observed. This could be explained based on interactions between the APIs (Cur and Pio) and CS during preparation, using TPP as a cross-linker agent. Although some diffraction peaks were still present, an amorphous state could be attributed to the CS-APIs NPs.

Based on the X-ray analysis and the values of the physical parameters (PS, PI, and ZP), it can be appreciated that the formulation of the APIs as CS-based NPs improved the solubility and bioavailability of the APIs.

#### 3.1.4. Fourier-Transform Infrared Spectroscopy

In order to prove the loading of the APIs (Cur and Pio) into the polymeric matrix, FTIR spectroscopy was used.

**Figure 3:**
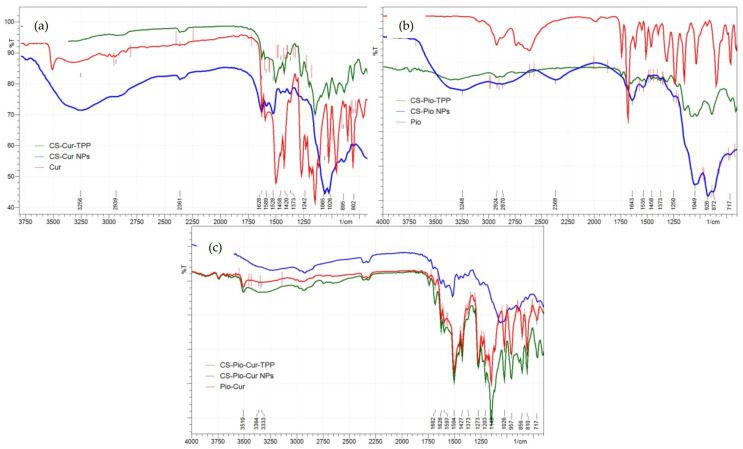
The FTIR spectra of CS-APIs NPs (CS-Cur (**a**), CS-Pio (**b**), and CS-Pio-Cur NPs (**c**) in comparison with APIs (Cur, Pio, and physical mixture of Pio-Cur) and physical mixtures of CS-APIs-TPP (CS-Cur-TPP, CS-Pio-Cur-TPP, and CS-Pio-Cur-TPP).

The analysis of the IR spectra of the APIs (Figure 3) revealed the characteristic absorption bands for Cur and Pio, in agreement with the literature data [20,26]. For Cur (Figure 3a), the characteristic absorption peaks were recorded at 1628 cm^−1^ (stretching vibration of the C=C and C=O of the inter-ring chain), 1597 cm^−1^ (symmetric stretching vibration of the aromatic ring C=C), 1528 cm^−1^ (C=O stretching vibration), 1420 cm^−1^ (alkenyl =C-H bending vibration), 1273 cm^−1^ (aromatic C-O stretching vibration), 1026 cm^−1^ (C-O-C stretching vibration), and 856 cm^−1^ (bending vibration of the CH of aromatic and exocyclic CH).

The specific absorption peaks for Pio (Figure 3b) were recorded at 1373 cm^−1^ (asymmetric CH_3_ vibration), 1504 cm^−1^ (CH_2_ vibration), 1535 cm^−1^ (asymmetric C=C stretching), and 1643 cm^−1^ (asymmetric C=O stretching), in agreement with the literature data [9]. For CS, the specific vibration of the pyranose units was recorded at 1065 cm^−1^.

The characteristic absorption peaks of Cur and Pio were also identified in the IR spectra of the CS-APIs NPs (CS-Cur NPs, CS-Pio NPs, and CS-Pio-Cur NPs) (Figure 3), which proved the loading of the APIs into the polymer matrix. For Cur, the characteristic peaks were shifted to 1582 cm^−1^ (C=C), 1520 cm^−1^ (C=O), 1065 cm^−1^ (C-O-C), and 887 cm^−1^ (CH), while for Pio, the characteristic peaks were shifted to 1504 cm^−1^ (CH_2_), 1528 cm^−1^ (C=C), and 1628 cm^−1^ (C=O). These recorded shifts could indicate that some interactions between the APIs and CS occurred in the CS-Pio-Cur NPs.

#### 3.1.5. Entrapment Efficiency (EE) and Loading Capacity (LC)

**Figure 4:**
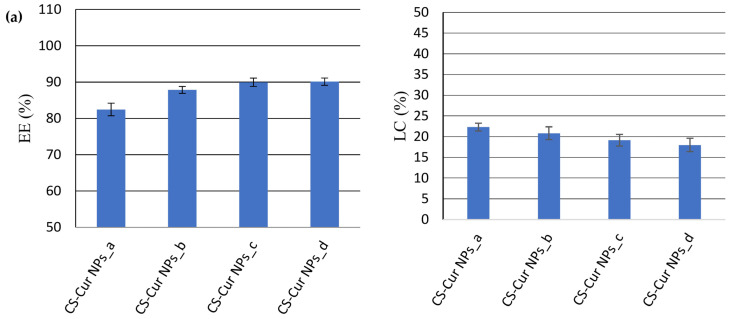

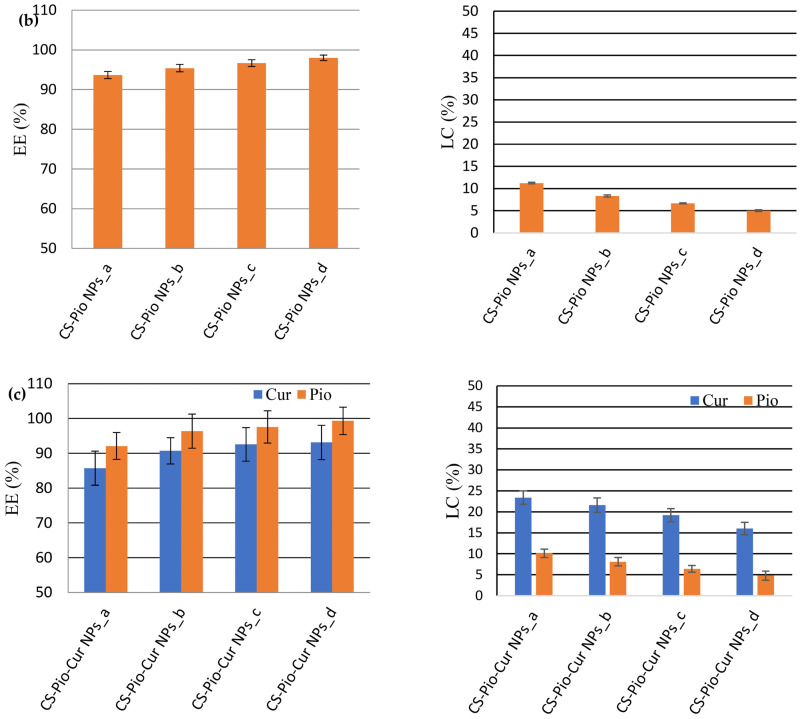
The effect of APIs’ concentration on EE (%) and LC (%) for CS-Cur NPs (**a**); CS-Pio NPs (**b**); and CS-Pio-Cur NPs (**c**) (*p* < 0.05).

The analysis of the results (Figure 4) revealed that the EE (%) decreased with the concentration of the APIs, while the LC (%) increased with it.

For the CS-Cur NPs (Figure 4a), the EE of Cur varied from 82.46% ± 1.74 (CS-Cur NPs_a) to 90.11% ± 1.01 (CS-Cur NPs_d), while the LC value decreased from 22.31% ± 0.94 (CS-Cur NPs_a) to 18.01% ± 1.61 (CS-Cur NPs_d). The most proper CS-Cur NP formulation seemed to be CS-Cur NPs_a, for which proper EE and LC values were recorded (EE = 82.46% ± 1.74; LC = 22.31% ± 0.94).

In the case of the CS-Pio NPs, the optimal formulation was also CS-Pio NPs_a, for which the EE was 93.67% ± 0.89 and the LC was 11.24% ± 0.17 (Figure 4b). In addition, it was observed that Pio seemed to increase a little bit the EE of Cur when both APIs were present in the same formulation (CS-Pio-Cur NPs). In the case of these last formulations (Figure 4c), the results support that the optimal formulation was CS-Pio-Cur NPs_a (0.6 mg Pio/1.5 mg Cur), for which the highest EE and LC values were recorded (EE: Cur—85.76% ± 4.89 and Pio—92.16% ± 3.79; LC: Cur—23.40% ± 1.62 and Pio—10.14% ± 0.98).

### 3.2. The API Release Study

#### 3.2.1. Development and Validation of HPLC Method

The developed HPLC method was validated according to the bioanalytical guidelines [27].

**Figure 5:**
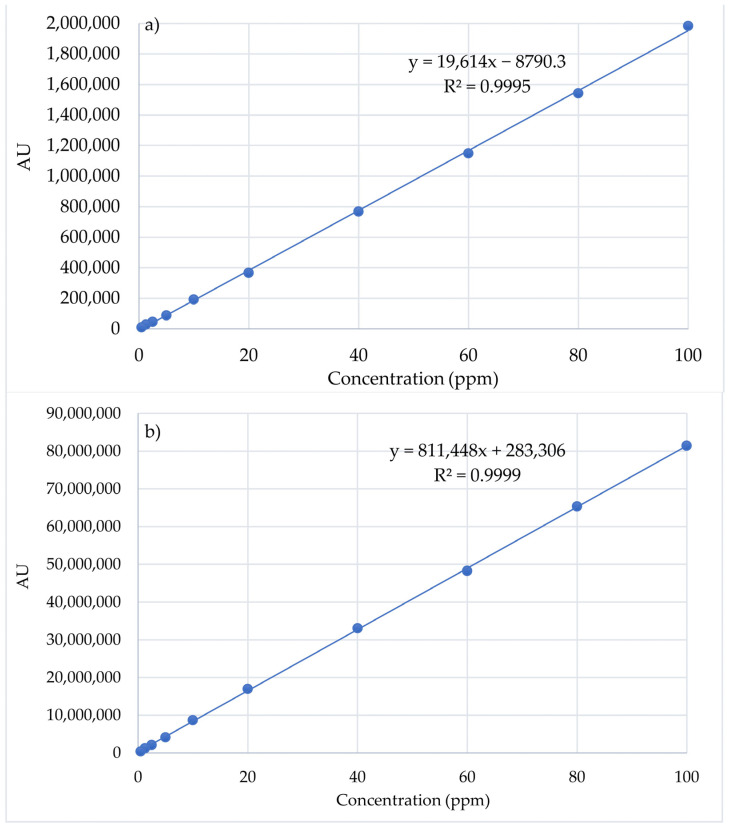
Calibration curves of Pio (**a**) and Cur (**b**) standards (n = 3).

The linearity of the method was studied in a 0.5–100 ppm concentration range for Cur and Pio. The average value of the AU was calculated for each concentration and was plotted in relation to the concentration (Figure 5). Statistical analysis of the results led to regression line equations. The linear regression data for the calibration curves of the APIs showed a good linear relationship across the concentration range.

The accuracy of the method was calculated based on the experimental HPLC data and the regression curve equation. It was noted that recovery for Pio was between 98.34% and 101.96% (100 ± 2%), while for Cur, it was between 99.5 and 101.43%.

The precision of the method could be expressed as the coefficient of variation (CV) or the relative standard deviation (RSD). The obtained results support the reproducibility of the method. The RSD (%) values were less than 2%, which confirms that the method has a high degree of accuracy and precision and, so, can be applied for quantitative measurements of the release of Cur and Pio from the developed CS-APIs NPs.

The results strongly support that the developed HPLC method has good sensitivity, precision, and accuracy, and can be applied for the simultaneous determination of the release of Cur and Pio from CS-APIs NPs.

#### 3.2.2. In Vitro API Release

**Figure 6:**
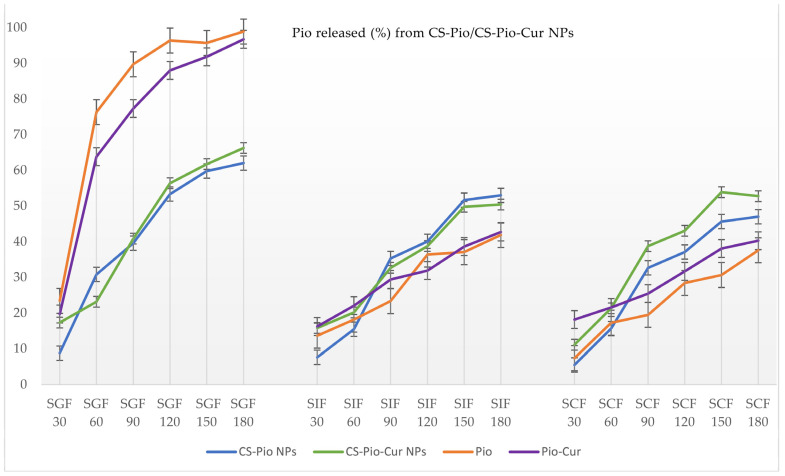
The percentage (%) of Pio released from CS-Pio NPs and CS-Pio-Cur NPs at different times and in different simulated fluids.

**Figure 7:**
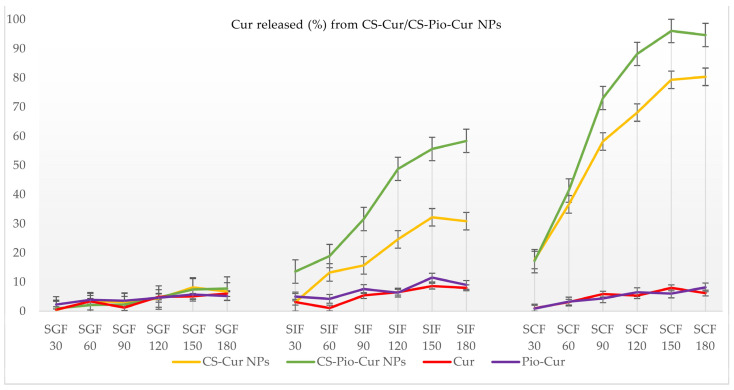
The percentage (%) of Cur released from CS-Cur NPs and CS-Pio-Cur NPs at different times and in different simulated fluids.

The release profiles of the APIs (Pio and Cur) from the CS-APIs NPs (CS-Pio/CS-Cur/CS-Pio-Cur NPs) using different simulated fluids (SGF, SIF, and SCF) at different times are presented in Figure 6 and Figure 7. In addition, the solubility and behavior of the APIs (Pio, Cur, and the physical mixture of Pio-Cur) in similar experimental conditions were also studied, and the data are presented for comparison.

*Pio released from CS-APIs NPs*

Using two-way ANOVA tests, it was found that the Pio release was statistically significantly (*p* < 0.05) influenced by time, which means it increased with the exposure time in the different simulated fluids. No statistically significant difference between CS-Pio and the CS-Pio-Cur NPs (F(1, 34) = 0.252, *p* = 0.619) was noted, which means that the Pio release is not influenced by the presence of Cur in the CS-Pio-Cur NPs. One-way ANOVA established no statistically significant difference between SGF, SIF, and SCF (F(2, 33) = 1161; *p* = 0.326) regarding the released Pio from CS-Pio and the CS-Pio-Cur NPs.

Pio is considered a weakly basic drug, with poor solubility in water (0.015 mg/mL) [28]. As expected, the quantified Pio as an API was higher in SGF (pH 1.6; 97.79 ± 3.01%) than in SGF (pH 7.00; 42.71 ± 2.78%) and SCF (pH 7.4; 40.24 ± 3.80%) at the end of the experiment (180 min).

In similar conditions, the quantified Pio released from the CS-APIs NPs was less in SGF but higher in SIF and SCF than the Pio as an API, which supports its formulation as NPs.

Furthermore, whether there was a statistically significant difference (*p* < 0.05) between the CS-Pio/CS-Pio-Cur NPs and APIs (Pio and Pio-Cur) was studied, and no statistically significant difference was noted.

*Cur released from CS-APIs NPs*

The release profiles of Cur from CS-Cur and the CS-Pio-Cur NPs using different simulated fluids (SGF, SIF, and SCF) at different times are presented in Figure 7. For comparison, the release profiles of Cur and the physical mixture of Pio-Cur in similar experimental conditions are also presented.

The analysis of the recorded results revealed that the value of Cur released (%) from the CS-based NPs (CS-Cur and CS-Pio-Cur NPs) was statistically significantly influenced (F (6, 60) = 9.459; *p* < 0.05) by the simulated fluids. More exactly, Cur was released in a higher percentage from CS-Cur and the CS-Pio-Cur NPs than Cur from the Cur-Pio physical mixture, especially in SIF (*p* = 0.002) and SCF (*p* = 0.000). No difference was observed for SGF (*p* = 0.765).

In the case of Cur and the Pio-Cur physical mixture, the quantified Cur was less than 10%, which is explained based on its low solubility in SIF, SGF, and SCF. Cur is a lipophilic polyphenol, insoluble in water, acid, and neutral pH solutions but soluble in alkaline media [29,30].

In similar experimental conditions, the value recorded for Cur released from the CS-Cur-NPs was much higher than pure Cur, explained by the CS-based formulation. For example, in SCF, the value recorded at 180 min was 80.34 ± 4.20% in comparison with the value of 6.20 ± 2.40% recorded for Cur. In addition, the presence of Pio improved the kinetic profile of Cur released from the CS-Pio-Cur NPs. The value recorded for Cur released from the CS-Pio-Cur NPs was 94.67 ± 3.90%, higher than the value of 80.34 ± 4.20% recorded for the CS-Cur NPs in SCF at 180 min.

As general remarks, the in vitro API (Cur and Pio) release assay showed that the developed co-delivery nanosystem, CS-Pio-Cur NPs, assures a controlled and prolonged release of Pio and Cur from the polymer matrix along the GI tract.

## 4. Conclusions

In order to target optimal hypoglycemic control and oxidative stress, which are involved both in the onset and progression of T2DM, a new co-delivery nanosystem, CS-Pio-Cur NPs, was prepared. To find the optimal formulation conditions (the CS concentration and the Cur-Pio ratio), simple nanosystems (CS-Cur NPs and CS-Pio NPs) were also developed. The developed CS-APIs NPs (CS-Pio-Cur NPs, CS-Cur NPs, and CS-Pio NPs) were fully characterized in terms of their particle size, polydispersity index, and zeta potential.

The XRD study demonstrated that the APIs (Cur and Pio) loaded into the CS NPs were in an amorphous state, which could be an important advantage because their solubility and bioavailability could be improved in comparison with standard APIs that are in a crystalline state.

The analysis of the FT-IR spectra supported the successful loading of the APIs into the CS matrix. In addition, some shifts in the characteristic peaks of the APIs were observed, which indicated that some interactions between the APIs and CS occurred in the CS-Pio-Cur NPs.

The HPLC method developed for the quantification of the APIs’ (Cur and Pio) release from the co-delivery nanosystem has high sensitivity, precision, and accuracy and was successfully applied in the release study. Based on the in vitro release of the APIs in the different simulated fluids (SGF, SIF, and SCF), it can be concluded that the developed CS-APIs NPs are suitable for oral administration, showing good absorption in the gastrointestinal tract. According to the obtained results, the developed nanoformulations can be potential candidates for multitarget antidiabetic therapy.

## Figures and Tables

**Table 1:** The composition of SGF, SIF, and SCF used as in vitro digestion model.

Component	SGF	SIF	SCF
Sodium taurocholate	0.0086 g	0.32 g	1.074 g
Lecithin	0.00308 g	0.032 g	0.488 g
Pepsin	0.02 g	-	-
Maleic acid	-	0.44 g	0.664 g
Sodium chloride	0.3998 g	0.802 g	1.70 g
Sodium hydroxide	-	0.28 g	0.42 g
Sodium oleate	-	-	2.43 g
Hydrochloric acid	pH 1.6	-	-
Distilled water	200 mL	200 mL	200 mL

**Table 2:** The effect of APIs’ concentration (Pio and Cur) on physical parameters of CS-APIs NPs (*p* < 0.05). APIs’ concentrations: CS-Cur NPs (a: 1.5 mg, b: 1.3 mg, c: 1.2 mg, and d: 1.0 mg), CS-Pio NPs (a: 0.6 mg, b: 0.5 mg, c: 0.4 mg, and d: 0.3 mg), and CS-Pio-Cur NPs (a: 0.6–1.5 mg, b: 0.5–1.3 mg, c: 0.4–1.2 mg, and d: 0.3–1.0 mg).

CS-APIs NPs	APIs	PS (nm)	PI	ZP (mV)
CS-Cur NPs	a	226.1 ± 7.1	0.150 ± 0.04	26.33 ± 0.40
	b	215.5 ± 4.8	0.262 ± 0.05	29.92 ± 0.21
	c	219.4 ± 6.3	0.277 ± 0.06	21.83 ± 0.25
	d	211.6 ± 6.0	0.104 ± 0.03	29.33 ± 0.46
CS-Pio NPs	a	228.1 ± 5.3	0.252 ± 0.05	25.01 ± 0.57
	b	247.1 ± 6.1	0.289 ± 0.05	29.48 ± 0.62
	c	245.1 ± 5.8	0.261 ± 0.03	23.91 ± 1.05
	d	230.1 ± 5.0	0.211 ± 0.04	23.76 ± 0.71
CS-Pio-Cur NPs	a	337.4 ± 6.2	0.268 ± 0.03	29.03 ± 0.51
	b	325.6 ± 7.0	0.266 ± 0.04	31.42 ± 0.48
	c	291.7 ± 6.5	0.277 ± 0.03	30.71 ± 0.31
	d	255.7 ± 6.3	0.273 ± 0.05	32.64 ± 0.39

## Data Availability

The original contributions presented in the study are included in the article, further inquiries can be directed to the corresponding author.
